# Knowledge, beliefs and intentions of African men in the Free State about prostate cancer screening

**DOI:** 10.4102/hsag.v27i0.2081

**Published:** 2022-12-05

**Authors:** Matthew O.A. Benedict, Wilhelm J. Steinberg, Frederik M. Claassen, Nathaniel Mofolo, Cornel van Rooyen

**Affiliations:** 1 Department of Family Medicine, Faculty of Health Sciences, University of the Free State, Bloemfontein, South Africa; 2 Department of Urology, Faculty of Health Sciences, University of the Free State, Bloemfontein, South Africa; 3 School of Clinical Medicine, Faculty of Health Sciences, University of the Free State, Bloemfontein, South Africa; 4 Department of Biostatistics, Faculty of Health Sciences, University of the Free State, Bloemfontein, South Africa

**Keywords:** knowledge, screening, intentions, African men, culture, beliefs, prostate cancer

## Abstract

**Background:**

African men are less likely to participate in prostate cancer (PCa) screening, which may be beneficial to some of them. Gaps in knowledge, cultural factors and beliefs are associated with their screening intentions.

**Aim:**

To determine the knowledge, cultural factors and screening intentions of African males regarding PCa screening.

**Setting:**

The study was conducted among African men attending randomly selected primary healthcare clinics in the Free State province.

**Methods:**

An analytical, cross-sectional survey using self-administered questionnaires developed in line with the Theory of Planned Behaviour constructs.

**Results:**

Of the 389 respondents, 18.3% had ever been screened for PCa with prostate-specific antigen (PSA) testing and 6.2% by digital rectal examination (DRE). About a quarter (24.4%) of the respondents had knowledge scores ≥ 50%. Factors associated with greater intent to screen for PCa were lower degree of fear/apprehension of PCa screening (mean score = 2.03; *p* < 0.001), higher perceived benefits of PCa screening (mean score = 2.69; *p* = 0.002), lower perceived situational barriers to PCa screening (mean score = 2.03; *p* = 0.006) and higher perceived risk of getting PCa (mean score = 2.66; *p* = 0.024).

**Conclusion:**

The observed low level of knowledge and practice of PCa screening among the respondents could be enhanced through PCa awareness strategies targeted at these men or those that could influence their decision making, especially healthcare providers. Factors that enhance screening intentions should be promoted.

**Contribution:**

This study improves on the scarce literature on factors associated with African men’s PCa screening intention.

## Introduction

Prostate cancer (PCa) remains a substantial healthcare burden. The global incidence of PCa was the second highest after lung cancer in 2020 (Sung et al. 2021). In 2020, the age-standardised incidence rate for PCa in South Africa was 68.3 per 100 000 men (World Health Organization, International Agency for Research on Cancer 2021). Globally, there were about 1 414 000 new PCa cases and 375 304 deaths in 2020 (Wang et al. 2022).

In South Africa, PCa is the leading neoplasm among men, with an incidence rate of 68 per 100 000 men in 2018 (Cassim et al. 2020). Prostate cancer accounts for about 13% of male deaths from cancer in South Africa (Babb et al. 2020). Though the aetiology of PCa is currently unknown, its non-modifiable risk factors include ageing, African ancestry, genetic factors and family history (Hayes & Bornman 2018; Rawla 2019). Both international (Jiang, Narayan & Warlick 2018; Siegel et al. 2020) and local (Dewar et al. 2018; Mofolo et al. 2015; Tindall et al. 2014) studies have shown racial disparities in PCa presentation (stage at presentation, prognosis and mortality), with men of African ancestry having the worst outcomes. Poor socio-economic status, educational level and lack of knowledge of the disease symptoms are some of the factors responsible for late presentation among South African men (Mofolo et al. 2015).

Prostate cancer screening with prostate-specific antigen (PSA) is controversial because of the associated overdiagnosis and overtreatment. However, more recent studies reveal that the net benefit of PSA screening may be beneficial to black men compared with the general population (Basourakos et al. 2022; US Preventive Services Task Force et al. 2018).

The South African Prostate Cancer Diagnostic and Treatment guidelines recommend PSA testing for black men 40 years and older (45 years and older for men of other races) with a life expectancy of more than 10 years if they have a family history of prostate or breast cancer in a first-degree relative, lower urinary tract symptoms and/or clinical suspicion of PCa, regardless of age group (Segone et al. 2013).

African men are less likely to participate in PCa screening (Kinyao & Kishoyian 2018), which may benefit some of them (Segone et al. 2013; Tracy, Brooks & Said 2021). Their attitude towards screening may be because of gaps in knowledge, cultural factors and beliefs (Bugoye et al. 2019; Mofolo et al. 2015). In a Kenyan study, family influence was found to be significantly associated with PCa screening intent among men (Mutua, Pertet & Otieno 2017).

Despite good knowledge of PCa among men in Central America, they had a poor perception of screening for the disease owing to the fear of the procedure and of receiving positive PCa result (Husaini et al. 2021).

Strong beliefs in the benefits of PCa screening, notwithstanding, some Kenyan men do not perceive men over 40 years at risk of getting PCa. Furthermore, they had relatively high fatalistic beliefs, a high degree of fear and a high level of influence of family members towards PCa screening (Mutua et al. 2017).

In a Tanzanian study, despite the respondents’ knowledge about PCa screening and their perception of being at risk of the disease, less than 8% had ever been screened (Bugoye et al. 2019). Likewise, a low PCa screening rate (5%) was found among Kenyan men in a study, although their intention to screen was high; the main barrier to PCa screening was their belief that they were well (Mbugua, Oluchina & Karanja 2012). Other possible barriers to PCa screening, as found in a Nigerian study, were ignorance of the disease, fear of a positive result and financial constraints (Ugochukwu et al. 2019).

In summary, men’s intentions towards PCa screening depend on their socio-economic status, the level of knowledge of the disease, culture and beliefs, and socially influential people in their lives.

Behavioural theories and models such as the theory of planned behaviour (TPB), theory of reasoned action (TRA), socio-ecological model (SEM) and health belief model (HBM) have engaged in understanding patients’ behaviours and decisions regarding health practices. The present study adopts the TPB to achieve the study objectives (Raingruber 2016).

According to Ajzen and Fishbein (1980), the TPB proposes that the most significant predictors of people’s performance of a behaviour are their behavioural intentions concerning its performance. Intentions, in turn, are predicted by three variables: attitudes, subjective norms and perceived behavioural control. Attitude refers to people’s overall evaluation of their performing the behaviour. Subjective norm refers to perceptions of social pressure from significant others to perform the behaviour. Perceived behavioural control refers to people’s appraisals of their ability to perform a behaviour (Sheeran, Conner & Norman 2001).

Research providing a theoretical approach to PCa screening behaviours among African men in South Africa is currently scarce.

### Aim

This study aimed to determine the knowledge, cultural beliefs and screening intentions of African men in the Free State regarding PCa screening. The primary objectives were to determine (1) the background characteristics of African men in the Free State, (2) their level of knowledge and the knowledge gaps regarding PCa and PCa screening, (3) their culture and beliefs regarding PCa screening and (4) their screening intentions. The secondary objective was to determine the factors that impact their screening intentions for PCa.

## Methods

### Study design

This study was an analytical cross-sectional survey of African men attending selected primary healthcare (PHC) clinics.

### Target population

The target population of the study included African men aged 40–70 years in the Free State.

Extrapolating from data available from StatsSA 2019 (Department of Statistics South Africa 2019), there are about 250 369 African men ≥ 40 years old in the Free State.

#### Sample size calculation and sampling

Using the Raosoft sample size calculator (Raosoft 2004), setting the margin of error at 5%, confidence level at 95%, response distribution at 50% and with the population size of 250 369, the sample size generated was 384.

The Free State is divided into five district municipalities, each having an average of 46 fixed PHC clinics (a total of 231). It was impractical to visit all the clinics because of financial and time constraints. The list of these clinics was obtained from the respective district offices. Through stratified simple random sampling method, 115 of these 231 fixed PHC clinics were selected (i.e. 23 clinics per health district). Four respondents were targeted per clinic, giving a total of 460 respondents targeted.

#### Exclusion criteria

To avoid introduction of bias because of prior knowledge, those already diagnosed with PCa or benign prostatic hyperplasia and those who have had prostate investigations, such as biopsy or ultrasound, were excluded from the study.

### Measurement, data collection and the questionnaire

A self-administered survey questionnaire was used. With the assistance of language translators from the University of the Free State (UFS), the questionnaire was translated from English into Sesotho and Isizulu, which are the common local languages (Wazimap 2016) in the area. The research assistant, fluent (spoken and written) in these three languages, helped some respondents clarify questions.

The questionnaire adopted for this study was a validated modified version of the Thomas Jefferson University Prostate Cancer Screening Survey Questionnaire, which was developed using the constructs of the TPB (Kenerson 2010; Mutua et al. 2017).

The questionnaire consisted of six sections: Sections A - F.

Section A (Table 3) contained demographic and background details: age, ethnic group, level of education, occupation, relationship status, residential area, medical aid cover, family history of PCa, previous PCa screening, perceived risk of getting PCa and preferred healthcare provider.

Section B (Table 5) contained the knowledge items. There were 16-point (one point each) knowledge-testing statements with *True, False* or *I don’t know* options.

Bloom’s cut-off points (Yimer et al. 2014) were used to categorise the knowledge levels as follows:

- good knowledge (80% – 100% correct responses)
- moderate knowledge (60% – 79% correct responses)
- poor knowledge (< 60% correct responses).

Section C consisted of the eligibility criteria for participation in the PCa screening survey. To be eligible, the respondents must: (1) be 40–70 years of age, (2) be African by race, (3) have heard about PCa, (4) not have been previously or currently diagnosed with PCa and (5) not have had a prostate biopsy in the past.

Section D was the PCa screening survey. This section explored the cultural factors (fatalism, fear/apprehension, perceived benefits, subjective norms, situational barriers and certain contributory factors) likely to be associated with respondents’ intent to screen for PCa. Total subscale scores were generated for fatalism, fear/apprehension, perceived benefits and subjective norms before statistical inferences were tested.

Section E explored the respondents’ source(s) of knowledge about PCa and PCa screening, while Section F checked the respondents’ perceived need for more knowledge about PCa and PCa screening and their preferred method of receiving such knowledge.

#### Constructs and variables contained in the questionnaire

Constructs of the TPB were operationalised to examine the role of social and cultural determinants of PCa screening behaviours among African men.

#### Attitude towards prostate cancer screening

This is the degree to which PCa screening is positively or negatively valued. Attitude and beliefs were operationalised by measuring fatalistic views of PCa, PCa screening, fears/apprehension and the perceived benefits of PCa screening.

#### Subjective norms and prostate cancer screening

This represents perceived social pressure to adhere to PCa screening. Normative beliefs were operationalised by measuring the social influence of relevant other(s) (healthcare providers and family members) on PCa screening behaviours.

#### Situational barriers to prostate cancer screening

Situational barriers are factors that hinder the decision to participate in PCa screening. The situational barriers assessed in this study included concerns about cost, time commitment, embarrassment and pain associated with PCa screening.

#### Prostate cancer screening intent

The intent was operationalised by measuring the intention to participate in PCa screening within a six-month period.

#### Contributory or demographic factors

These are factors that may or may not influence the intention to participate in PCa screening tests. Such factors include demographic characteristics, PCa screening history, family history (first-degree relatives) of PCa, perceived risk of PCa and socio-economic status of respondents.

Items relating to attitude, subjective norms, situational barriers, screening intent and contributory or demographic factors as listed above were measured on a four-point Likert scale (1 *= strongly disagree*, 2 = *sort of disagree*, 3 = *sort of agree* and 4 = *strongly agree*).

#### Prostate cancer knowledge

The knowledge construct was added to the TPB model to assess the relationship between understanding PCa and associated beliefs about PCa and PCa screening. Knowledge was operationalised by measuring the knowledge of symptoms of PCa, risk factors for PCa, screening age guidelines and side effects from PCa treatment.

#### Reliability and validity of the questionnaire

Questions similar to this study had been tested in a study conducted among African-American men (Kenerson 2010). The internal consistency (Cronbach’s α) for the subscale scores in the study was 0.76, 0.67, 0.78, 0.70 and 0.95 for fatalism, fear/apprehension, perceived benefits, subjective norms and screening intent, respectively.

Items on PCa knowledge scale measured respondents’ knowledge of PCa screening limitations, signs and symptoms, risk factors and guidelines. Each item required a *true, false* or *I don’t know* response. The reliability using factor analysis was 0.61. Construct validity was based on factor analysis and factor loading of 0.35 or greater. Cronbach’s α was 0.77.

The interpretations of the subscale scores are described in Table 1. Table 2 shows the level of impact for subscale scores.

**TABLE 1:** Subscale scores interpretation.

Culture and beliefs	Interpretation of subscale score	
	Higher score	Lower score
Fatalism	Negative attitude/valued beliefs	Positive attitude/valued beliefs
Fear/apprehension	Negative attitude/valued beliefs	Positive attitude/valued beliefs
Perceived benefits	Positive attitude/valued beliefs	Negative attitude/valued beliefs
Subjective norms	Greater perceived social influence for PCa screening	Less perceived social influence for PCa screening
Situational barriers	Greater number of barriers or less perceived behavioural control	Less number of barriers or less perceived behavioural control
Screening intent	Greater intent to go for PCa screening	Less intent to go for PCa screening
Contributory factors	Higher perceived risk of getting PCa	Lower perceived risk of getting PCa

*Source*: Adapted from Kenerson, D., 2010, ‘Use of theory of planned behaviour to assess prostate cancer screening intent among African American men’, dissertation, School of Nursing, Vanderbilt University, Nashville, TN

PCa, prostate cancer.

**TABLE 2:** Subscale score (four-point Likert scale) and corresponding level of impact.

Subscale score	Impact
< 1.5	No impact
≥ 1.5 but < 2.5	Little or low impact
≥ 2.5 but < 3	Moderate impact
≥ 3	High impact

*Source*: Adapted from Von Pressentin, K.B., Mash, R.J., Baldwin-Ragaven, L., Botha, R., Govender, I., Steinberg, W.J. et al., 2018, ‘The perceived impact of family physicians on the district health system in South Africa: A cross-sectional survey’, *BMC Family Practice* 19(1), 24. https://doi.org/10.1186/s12875-018-0710-0

### Pilot study

The questionnaire was pre-tested on 20 respondents attending one of the selected PHC clinics for other non-PCa-related reasons. These respondents were chosen in succession. The purpose of the pilot test was to ensure that the questions were balanced and correctly constructed and that the crucial information would be obtained. The 20 piloted questionnaires were included in the study as no significant changes arose from the pilot study.

### Data analysis

The data were analysed by the Department of Biostatistics, Faculty of Health Sciences, UFS, using SAS Version 9.3 (SAS Institute Inc., Cary, North Carolina, United States of America). Descriptive statistics (e.g. median and standard deviation [SD]) were used for continuous variables, while frequencies and percentages were computed for categorical data. Association between variables was assessed using chi-squared or Fisher’s exact tests. A *p*-value of < 0.05 was taken to be significant.

### Ethical considerations

This study was approved by the Health Sciences Research Ethics Committee of the Faculty of Health Sciences, UFS, with ethical clearance number: UFS-HSD2020/1481/2411. Permission to conduct the study was obtained from the head of the Free State Department of Health.

Following a detailed description of the study, signed informed consent was obtained from each respondent before they participated in the study. The self-administered questionnaire was anonymous as no names of respondents were recorded on any of the documents.

## Results

### Background and socio-demographic characteristics of respondents

A total of 389 men participated out of the 460 eligible respondents invited, giving a response rate of 84.6%. Table 3 summarises the respondents’ background characteristics.

**TABLE 3:** Background and socio-demographic characteristics of respondents.

Variable	*n*	%
**Age (years)**		
40–49	225	57.8
50–59	112	28.8
≥ 60	52	13.4
**Ethnic group**		
Sesotho	186	47.8
Xhosa	81	20.8
Tswana	57	14.6
Zulu	37	9.5
Pedi	22	5.7
Venda	5	1.3
Other	1	0.3
**Level of education**		
Grade 12 (Matric)	109	28.0
Some secondary level (Grades 8–12)	101	26.0
Tertiary	61	15.7
Some primary level (Grades 1–7)	60	15.4
Primary level (Grade 7) completed	44	11.3
No formal education	14	3.6
**Medical aid**		
No	287	73.8
Yes	102	26.2
**Level of skill**		
Skilled	149	38.3
Unskilled	90	23.1
Semi-skilled	66	17.0
Never employed	50	12.9
Highly skilled and/or professional	34	8.7
**Source of income**		
Employed by an organisation	153	39.3
Self-employed	86	22.1
Government grant	78	20.1
No income	60	15.4
Retired (on pension)	11	2.8
Other	1	0.3
**Monthly household income**		
< R5 000	210	54.0
R5 000–R10 000	83	21.3
R10 000–R15 000	45	11.6
R15 000–R20 000	17	4.4
R20 000–R25 000	24	6.1
> R25 000	10	2.6
**Relationship status**		
Married	189	48.6
Single (never married)	76	19.5
Separated and/or divorced	52	13.4
Living as married (civil union)	38	9.8
Widowed	34	8.7
**District of residence**		
Mangaung	228	58.6
Thabo Mofutsayane	63	16.2
Lejweleputswa	39	10.0
Xhariep	32	8.2
Fezile Dabi	27	7.0
**Residential area**		
Rural	309	79.4
Urban	80	20.6
**1st degree family member with cancer?**		
No	316	81.2
Yes	73	18.8
**Top 1st degree family members with cancer (*n* = 63)**		
**Prostate cancer**		
Brother	3	4.8
Father	1	1.6
**Other cancers**		
Grandmother – breast cancer	14	22.2
Brother – lung cancer	13	20.6
Sister – breast cancer	13	20.6
Father – lung cancer	10	15.9
Grandfather – lung cancer	9	14.3

The median age of the respondents was 48 years (range 40–70 years). Only four of the respondents’ relatives had PCa (one father and three brothers).

Most (*n* = 188, 48.3%) of the respondents had never had a PSA test, 130 (33.4%) did not know about the test, 42 (10.8%) had the test over a year ago, while 29 (7.5%) had the test within the past one year. For the 71 respondents who had PSA testing in the past, their perception of the healthcare providers’ conduct of shared decision-making was assessed (Table 4).

**TABLE 4:** Respondents with previous prostate-specific antigen testing’s perception of healthcare providers’ conduct of shared decision-making (*n* = 71).

Shared decision-making criteria	Respondents’ perception of healthcare providers’ conduct					
	Never		Partially		Fully	
	*n*	%	*n*	%	*n*	%
The doctor or healthcare worker discussed the advantages of the screening blood test with me	9	12.7	27	38.0	35	49.3
The doctor or healthcare worker discussed the disadvantages of the screening blood test with me	48	67.6	12	16.9	11	15.5
The doctor or the healthcare worker told me some experts disagree about whether men should have prostate-specific antigen test or not	49	69.0	17	24.0	5	7.0

The majority (*n* = 207, 53.2%) of the respondents had never had a digital rectal examination (DRE), 158 (40.6%) did not know about the DRE, 16 (4.1%) had a DRE over a year ago, while eight (2.1%) had a DRE in the past one year.

Only 53 (13.6%) respondents felt that they were at risk of getting PCa.

The respondents’ preferred (first choice) healthcare providers were state PHC providers (*n* = 217, 55.8%), private general practitioners (*n* = 110, 28.3%), spiritual healers (*n* = 39, 10.0%) and traditional healers (*n* = 23, 5.9%).

### Assessment of respondents’ knowledge regarding prostate cancer screening

The mean knowledge score was 5.67 (range 0–14 points, ± standard deviation [SD] 2.88) out of 16 points. Based on the Bloom’s cut-off points, the majority of respondents (*n* = 355, 91.2%) had poor knowledge (i.e. scored < 60%). However, 95 (24.4%) scored ≥ 50%. Table 5 shows the respondents’ responses to the different knowledge statements.

**TABLE 5:** Respondents’ responses to knowledge statements.

Knowledge statement	Respondents’ response†					
	True		False		I don’t know	
	*n*	%	*n*	%	*n*	%
Men who have several family members (blood relatives) with prostate cancer are more likely to get prostate cancer	**202**	**51.9**	19	4.9	168	43.2
A man can have prostate cancer and have no problems or symptoms	**100**	**25.7**	138	35.5	151	38.8
Men < 40 years old are more likely to get prostate cancer	218	56.0	**63**	**16.2**	108	27.8
Difficulty in passing urine may be a symptom of prostate cancer	**149**	**38.3**	97	24.9	143	36.7
Certain diet or eating habits might increase one’s risk for developing prostate cancer	**106**	**27.3**	127	32.6	156	40.1
Obesity (being overweight) and cigarette smoking have no effect on prostate cancer risk	143	36.7	**115**	**29.6**	131	33.7
White men are at greater risk of developing prostate cancer	142	36.5	**115**	**29.6**	132	33.9
Frequent pain often in your lower back could be a sign of prostate cancer	**89**	**22.9**	135	34.7	165	42.4
Prostate cancer screening should be reserved for men over 70 years old	96	24.7	**160**	**41.1**	133	34.2
Some treatments for prostate cancer can make it harder for men to control their urine	**172**	**44.2**	96	24.7	121	31.1
Some treatments for prostate cancer can cause problems with a man’s ability to have sex	**207**	**53.2**	64	16.5	118	30.3
Prostate cancer can be cured if diagnosed early enough	**233**	**59.9**	51	13.1	105	27.0
Doctors can tell which men may die from prostate cancer and which men will not be harmed by prostate cancer	**75**	**19.3**	164	42.2	150	38.5
An abnormal prostate-specific antigen (PSA) blood test means I have cancer for sure	101	26.0	**88**	**22.6**	200	51.4
I can have cancer and have a normal PSA blood test	**161**	**41.4**	71	18.2	157	40.4
Prostate cancer may grow slowly in some men	**185**	**47.6**	46	11.8	158	40.6

PSA, prostate-specific antigen.

† , Values in bold indicate the correct answer for that specific statement.

### Assessment of respondents’ culture and beliefs about prostate cancer screening

The ‘fatalism’ mean score was 2.25 (± SD 0.68), indicating that these respondents held little fatalistic beliefs related to PCa and PCa screening. Examples of fatalism items include ‘If I am meant to get prostate cancer, I will get it no matter what I do’ and ‘If I get prostate cancer, nothing can be done to cure me of the disease’. The ‘fear/apprehension’ mean score was 2.03 (± SD 0.69), indicating a low degree of fear/apprehension associated with PCa and PCa screening among these respondents. Examples of fear/apprehension items include ‘I am bothered by the possibility that prostate cancer screening might be physically uncomfortable’ and ‘Men who go through prostate cancer screening will have more problems than men who do not go through screening’. The ‘perceived benefits’ of screening’s mean score was 2.69 (± SD 0.60), indicating moderate beliefs in the benefits of screening among these respondents. Examples of perceived benefit items include ‘I believe that going through prostate cancer screening would help me to be healthy’ and ‘I believe that I can protect myself from prostate cancer by going through screening’.

The ‘social influence’ mean score was 2.90 (± SD 0.72), showing a moderate perceived level of influence that family members and healthcare providers have on PCa screening among these respondents. Examples of subjective norm items include ‘I want to do what the doctor I see thinks I should do about prostate cancer screening’ and ‘Members of my immediate family are likely to think I should go through prostate cancer screening’.

The situational barrier mean score was 2.03 (± SD 0.64), indicating only a few barriers perceived among these respondents. Examples of situational barrier items include *‘*I have more important things to do than go for prostate cancer screening’ and ‘Going through prostate cancer screening would be embarrassing’.

The ‘contributory factor’ mean score was 2.66 (± SD 0.75), which indicates a moderate perceived risk of getting PCa among these respondents. Examples of contributory factor items include ‘I believe it is likely I will get prostate cancer at some time in the future’ and ‘I think African men are more likely to develop prostate cancer than white men’.

The majority of the respondents had multiple sources of knowledge regarding PCa, which included health education from doctors and nurses (52%), media (e.g. television [TV], radio and the Internet) (50%), literature (books, articles and newspapers) (26%), family members (19%) and friends (17%).

Most of the respondents (82.8%) felt the need for additional knowledge regarding PCa; aspects requiring additional knowledge were symptoms (78%), risk factors (76%), treatment (66%), the value of PSA (63%), the value of DRE (59%), diagnosis (56%) and the importance of certain nutrients in PCa prevention (48%). Methods suggested by the respondents for the dissemination of additional knowledge included informational leaflets or pamphlets (73%), talking to healthcare providers (e.g. doctors and nurses) (50%), radio (46%), social media (e.g. Facebook and WhatsApp) (46%), home visits by healthcare providers (e.g. the community health workers) (45%), television (41%) and posters in public spaces (26%).

### Prostate cancer screening intentions

The ‘screening intent’ mean score was 2.88 (± SD 0.70), reflecting a moderate level of intention to screen for PCa among these respondents. Examples of screening intent items include ‘I intend to have a prostate cancer screening test (prostate-specific antigen blood test) in the next 6 months’ and ‘In the next 6 months, I plan to discuss prostate cancer screening with a doctor’.

### Factors associated with prostate cancer screening intentions

The relationship between certain background characteristics, cultural factors and beliefs, knowledge and the intention to screen for PCa was assessed using the chi-square test, as shown in Table 6.

**TABLE 6:** Bivariate analysis of attitude and beliefs about prostate cancer versus intention to screen.

Independent variable	Strong intention (*n* = 354)		Weak intention (*n* = 35)		*p*
	*n*	%	*n*	%	
**Fatalistic belief**					0.182
Strong	268	75.7	30	85.7	
Weak	86	24.3	5	14.3	
**Fear/apprehension**					< 0.000*
Great	176	49.7	29	82.9	
Less	178	50.3	6	17.1	
**Perceived benefit**					0.002*
Great	338	95.5	28	80.0	
Less	16	4.5	7	20.0	
**Social influence**					0.375
Strong	320	90.4	30	85.7	
Weak	34	9.6	5	14.3	
**Situational barrier**					0.006*
Great	157	44.4	24	68.6	
Less	197	55.6	11	31.4	
**Perceived risk**					0.024*
Strong belief	304	85.9	25	71.4	
Weak belief	50	14.1	10	28.6	

* , Statistically significant association.

The following factors were associated with greater intent to go for PCa screening: lower degree of fear/apprehension of PCa screening (*p* < 0.001), higher perceived benefits of PCa screening (*p* = 0.002), lower perceived situational barriers to PCa screening (*p* = 0.006) and higher perceived risk of getting PCa (*p* = 0.024).

The results showed no statistically significant associations between the level of knowledge regarding PCa, fatalistic beliefs, subjective norms, socio-demographic and background variables and PCa screening intent.

## Discussion

This study examined the knowledge, cultural factors and belief and screening intentions of African men in the Free State regarding PCa screening. Factors associated with the respondents’ screening intentions for PCa were also examined.

### Socio-demographic characteristics

The median age of respondents was 48 years (range 40–70 years). In a similar Kenyan study, the mean age was 49.8 (± SD 16.7) years (Mutua et al. 2017). Men at lower risk for PCa (40–49 years) were more represented in this study, which may indicate the distribution of health-seeking behaviour among men in the study setting. This is corroborated by a similar study by Mofolo et al. (2015).

Almost half of the respondents (48.6%) were married, 69.7% had at least secondary level education and 35.5% had no income or depended on a government grant. These socio-demographic characteristics seem typical of the study setting, as corroborated by findings of a similar study where 64% of the respondents were married, 53.2% had at least secondary level education and 30% were unemployed (Mofolo et al. 2015).

### Background characteristics relating to prostate cancer screening

Less than a fifth (18.3%) and less than a tenth (6.2%) of the respondents had ever done a PSA test and DRE, respectively, in the past. This may imply a low level of risk perception and the lack of screening opportunities in the community as the respondents included in this study have at least heard about the disease. In the study setting, there may also be the need to reiterate health education on this disease. In a similar study, only 2.4% of the respondents had ever done a PSA test in the past (Mofolo et al. 2015). Also, in a Ghanaian study, only 10% of the respondents had ever done a PSA test (Yeboah-Asiamah et al. 2017). Studies have shown a lack of awareness, fear of a positive result, the discomfort of DRE and financial constraint as possible reasons for low screening rates (Husaini et al. 2021; Ugochukwu et al. 2019; Yeboah-Asiamah et al. 2017). On the other hand, there may be knowledge gaps among healthcare providers and subsequent poor awareness of the disease in the community. There is, therefore, the need to determine and address the factors associated with this low screening rate in the present study setting. The reason for a relatively higher screening percentage in this study may be because ‘having previously heard about prostate cancer’ was one of the eligibility criteria to participate.

Only 53 (13.6%) respondents felt they were at risk of getting PCa, whereas others felt either they were not at risk or were unsure. This may explain the low screening rate found in this study, as explained above. This is unlike a similar study conducted in Kenya, where 64% of the respondents thought they might be at risk of getting the disease (Mutua et al. 2017). The low perceived risk of getting PCa in this study may be attributed to the poor knowledge of the disease.

As shown in Table 4, the respondents perceived their healthcare providers’ conduct of shared decision-making for PCa screening to be poor; therefore, it is unlikely that the PHC providers in the study setting are aware of the proper conduct of shared decision-making. Therefore, there is a need for these healthcare providers to be acquainted with the six steps engaged in the conduct of shared decision-making and be trained on how to practise these steps (Ng & Lee 2021).

About one-fifth (15.9%) of the respondents preferred spiritual or traditional healers as their first-line healthcare providers. This may be a reflection of the religious and cultural beliefs in the study setting. In a study by Chali, Hasho and Koricha (2021), 81.5% of the respondents practised traditional medicine use. Affordability, religious affiliation and distance from home were some reasons for their preference for traditional medicine. There is a need for collaboration with traditional healthcare providers to enhance their knowledge regarding PCa screening, counselling and early diagnosis.

### Respondents’ knowledge of prostate cancer screening

Even though ‘having previously heard about prostate cancer’ was one of the eligibility criteria for this study, less than one-quarter of the respondents (24.4%) scored ≥ 50%. This is unlike a similar study where a moderate to high level of knowledge was found among 79.1% of those who had previously heard of PCa (Mofolo et al. 2015). In another study from Cameroon, a medium level of knowledge was found among 55.2% of the respondents (Kaninjing et al. 2018). There may be the need to structure more detailed health education on this subject in the community, with frequent reiteration.

Respondents’ prior sources of knowledge regarding PCa included health education from healthcare providers, media (TV, radio and the Internet), literature (books, articles and newspapers), family members and friends. This finding is similar to a Kenyan study where respondents’ prior sources of knowledge were radio, health facility and healthcare provider and media (newspaper, magazine and TV) (Kinyao & Kishoyian 2018). Over 80% of the respondents felt they needed additional knowledge about PCa screening. Aspects requiring such additional knowledge were symptoms, risk factors, treatment, screening tests (PSA and DRE), diagnosis and the value of nutrients. Respondents’ preferred methods for knowledge delivery included informational leaflets and pamphlets, talking to healthcare providers (healthcare facilities and home visits), radio, social media (Facebook and WhatsApp), TV and public posters. Similarly, the preferred sources of information found in a Kenyan study were newspapers, TV, radio, website, community health workers, the hospital and through relatives (Kinyao & Kishoyian 2018). There is, therefore, the need to consider these various aspects as well as the preferred methods of knowledge delivery when developing health education strategies for the community.

### Respondents’ attitudes and beliefs about prostate cancer screening

The respondents in this study hold little fatalistic beliefs and fear/apprehension regarding PCa screening. They showed moderate beliefs in the benefits of PCa screening and a moderate perceived level of influence from family members and healthcare providers to go for screening. They indicated few barriers to PCa screening, and a moderate perceived risk of getting PCa. These findings partly align with those from a study among African-American men: the ‘fatalism’ mean score was 1.36, which indicates that this sample held relatively weak fatalistic beliefs related to PCa and PCa screening, and the ‘fear/apprehension’ mean score was 1.77, which shows a low degree of fear/apprehension associated with PCa and PCa screening. The respondents’ ‘perceived benefits’ of screening mean score was 3.58, representing strong beliefs in the benefits of screening. The ‘social influence’ mean score was 3.17, representing a high perceived level of influence family members and physicians had on PCa screening among this sample (Kenerson 2010).

The present study’s findings may be a reflection of a positive attitude towards health, which had been gradually inculcated into the respondents, as they are PHC users. In contrast, a Kenyan study showed that the respondents held relatively strong fatalistic beliefs (mean score of 3.8) and a high degree of fear/apprehension (mean score of 3.2). They, however, also had strong beliefs in the benefits of screening (mean score of 4.02), and a great influence of family members on screening (mean score of 3.8) (Kinyao & Kishoyian 2018). Of note is that a similar study conducted a year earlier in the same country yielded very similar results (Mutua et al. 2017).

### Respondents’ screening intentions and associated factors

The respondents in this study had a moderate level of intention to screen for PCa, which partially aligns with a study among African-American men where the ‘screening intent’ mean score was 3.01, reflecting a strong intention to screen for PCa among this sample (Kenerson 2010). The low screening rate in our study notwithstanding, the willingness of the respondents to screen may be indicative of a positive attitude and health-seeking behaviour.

The following factors were associated with greater intent to go for PCa screening: lower degree of fear/apprehension of screening, higher perceived benefits of screening, lower perceived situational barriers to screening and higher perceived risk of getting PCa. In an American study, perceived benefits of screening and social influence were associated with greater intent to screen for PCa (Kenerson 2010). However, in a Kenyan study, only fear had a statistically significant association with PCa screening intent (Kinyao & Kishoyian 2018).

The present study showed no associations of statistical significance between the level of knowledge regarding PCa, fatalistic beliefs, subjective norms and socio-demographic and background variables and PCa screening intent. Similar findings were observed in the Kenyan study regarding the non-association of these factors (Kinyao & Kishoyian 2018). Although the majority of the respondents in this study were married, the non-association of subjective norms and marital status with screening intention may imply the non-involvement of women in issues relating to men’s health in the study setting. Most of the respondents had attained at least secondary level education, yet this did not translate into greater screening intention. Factors other than the level of education may therefore determine screening intent among men in the study setting. The respondents in this study were users of PHC clinics, which are accessible and affordable; this may explain the reason for the non-association of income, residential area and medical aid status with screening intention.

Primary healthcare providers should be encouraged to continue promoting an attitude that will encourage greater screening intention among men. Prostate cancer health education should alleviate fear and barriers to screening, promote the benefits of screening and improve the awareness of PCa among men over the age of 40 years.

### Limitations

Because of the coronavirus disease 2019 pandemic, there were restrictions on the conduct of research at private PHC facilities. Hence, the respondents in this study were state PHC clinic users: the study findings may therefore not be generalisable. However, the state PHC facilities were randomly selected. Also, the use of self-administered questionnaires is not without flaws (e.g. forgetfulness), which may have a negative impact on the reliability of the results. However, the results may further contribute to the body of literature on knowledge, cultural factors (and belief) and screening intentions of African men regarding PCa screening, which is presently scarce in South Africa.

## Conclusions and recommendations

The respondents in this study have a low level of knowledge and practice of PCa screening. Both fatalistic belief and fear/apprehension towards PCa screening are low. The observed low level of knowledge and practice of PCa screening could be amenable to enhanced educational and awareness strategies, including clarification of cultural misconceptions regarding PCa screening – the respondents’ self-acclaimed knowledge gaps and preferred methods for knowledge delivery should be borne in mind. Factors shown to be associated with greater screening intent should be promoted. Appropriate strategies should also be directed at socially influential people in the lives of these men, including their preferred healthcare providers.

Lastly, the study findings should be communicated to PHC providers and other relevant stakeholders. Given the necessary support of the government and policymakers, early diagnosis and prevention of aggressive disease can be enhanced in the community through health education and awareness.
